# Pathogenesis of pepsin-induced gastroesophageal reflux disease with advanced diagnostic tools and therapeutic implications

**DOI:** 10.3389/fmed.2025.1516335

**Published:** 2025-02-19

**Authors:** Chong Li, Xiwen Cao, Hongxia Wang

**Affiliations:** ^1^Queen Mary College, Nanchang University, Nanchang, China; ^2^The Affiliated Hospital of Inner Mongolia Medical University, Hohhot, China

**Keywords:** gastroesophageal reflux disease, pepsin, pathogenesis, diagnosis, gastroenterology

## Abstract

Gastroesophageal reflux disease (GERD) is a common gastrointestinal disorder that significantly affects populations in both developing and developed countries. Due to both intrinsic pathology and extrinsic risk factors, the incidence of GERD has risen substantially in recent decades. This disorder results from an imbalance between the esophagus’s defensive mechanisms and the harmful effects of the refluxate. The pepsin, an enzyme secreted exclusively by the stomach, plays a critical role in the pathogenesis of GERD due to its invasiveness effects in acidic environments. By thoroughly understanding the pathogenesis of pepsin-induced GERD, we could better address its diagnostic and therapeutic potential in clinical practice. Although current diagnostic tools are widely used, they have several limitations. As a result, researchers have increasingly focused on the salivary pepsin test, a novel diagnostic method that utilizes the specific pathological mechanisms of pepsin. To overcome the drawbacks of the currently used salivary pepsin test, fluorescence response detection has been integrated with other technologies. Beyond its diagnostic significance, pepsin in saliva may also serve as a target for GERD management in innovative clinical trials. In this review, we summarize the latest advancements in the diagnosis and management of GERD to improve patient outcomes.

## 1 Introduction

As GERD is one of the most common gastrointestinal disorders, affecting approximately 15–25% of adults in developed countries and 10% in developing countries. It is primarily characterized by heartburn and regurgitation, though other symptoms such as dysphagia, persistent cough, and asthma may also occur (1–3). Beyond reducing quality of life, GERD is associated with an increased risk of complications, including esophagitis, esophageal strictures, esophageal ulcers, esophageal stenosis, and more severe conditions like Barrett’s esophagus and esophageal cancer (1).

Normally, there is a balance between the harmful effects of gastric refluxate on the esophageal lining and the esophagus’s defensive anti-reflux mechanisms. A breakdown in this balance, either by weakening the defense mechanisms or increasing erosive forces, can lead to pathogenetic alterations of GERD, encompassing esophageal exposure, resistance of the esophageal mucosal epithelium, and visceral sensitivity (4). Consequently, individuals with primary pathological factors such as malfunctioning anti-reflux barriers and impaired esophageal clearance and buffering would have more chance to be predisposed to GERD.

Though there exists various proven diagnostic methods for GERD, including specific questionnaires, anti-secretory inhibitors, endoscopy, and esophageal functional tests, they present notable limitations. Such as invasiveness, high cost, and low sensitivity and specificity. Recently, researchers are particularly drawn to pepsin, a protein implicated in the erosive degradation of the esophageal mucosa (5). As an acidic enzyme secreted solely by the stomach, the presence of pepsin in saliva is abnormal and can be used as a diagnostic marker for GERD. In addition to its diagnostic role, pepsin is also considered a therapeutic target and plays a significant role in GERD management (6).

The management of GERD can be categorized into two primary mechanisms: one aims to reduce reflux by suppressing gastric acid secretion, while the other focuses on strengthening the defensive function of the esophageal mucosal barrier to protect it from the erosive effects of gastric acid. Based on these strategies, GERD management typically involves two approaches: irreversibly inhibits pepsin activation and prevents the reactivation of pepsin in low pH environments after endocytosis uptake in late endosomes and trans-reticular Golgi apparatus (TRG) or use receptor antagonists on patients to block receptor-mediated pepsin uptake (7–9).

In this review, we summarize the pathological and potential molecular mechanism of pepsin-induced GERD, and gather the target molecules which play an important role in the progression of GERD and its subsequent tissue damage. The common diagnostic methods were elaborated and evaluated by summarizing the published basic research articles, clinical analysis articles and treatment guidelines issued by authoritative institutions. At the same time, we introduced the high specificity and sensitivity diagnostic method that combined fluorescence probe and new materials to be popularized in clinical practice, which we believe will be the wind vane for accurate diagnosis in the future. In addition, we focus on the current common clinical treatment methods and new compounds found in the latest research, which provide a more comprehensive direction and new thinking for further research on GERD in the future.

## 2 Pepsin-related pathological mechanism of GERD

The development and progression of GERD primarily depend on two factors: the invasive and destructive actions of pepsin and gastric acid, and the resistance and sensitivity of the epithelial barrier, which maintain the integrity of the esophageal mucosa. Pepsin exerts its effects by influencing various cellular signaling pathways, with its activity and sensitivity varying according to changes in pH and the environment. A compromised esophageal epithelial barrier, combined with increased sensitivity, increases the susceptibility of the mucosa to erosion. Together, these factors form the core pathogenic mechanisms of GERD.

### 2.1 Properties of pepsin

Pepsin is an aspartic protease derived from its precursor, pepsinogen, in acidic environments (10). In human gastric juice, the active sites of pepsin bind to substrates, initiating the process of proteolysis (10, 11). Through proteolysis, pepsin breaks down ingested proteins into small peptide fragments. Pepsin exhibits its highest activity at a pH of 2.0 and becomes inactive at a pH of 6.5, though it remains stable at a pH of 8.0. As a result, it can be reactivated when the pH drops again (12). Beyond its proteolytic function, pepsin plays a critical role as a major component of acidic refluxate (13).

These properties make pepsin an ideal biomarker for detecting reflux in clinical samples. However, its presence in saliva is often transient due to the episodic nature of reflux and the intermittent effects of swallowing, which can vary depending on food intake.

### 2.2 Pathological mechanisms of pepsin-induced GERD

The esophageal wall is composed of four layers: the mucosal layer, the submucosal layer, the muscular layer, and the adventitia. The mucosal layer, closest to the lumen, is responsible for neutralizing incoming acids and protecting the squamous epithelium of the esophagus from contact with refluxate (14). Under normal conditions, pepsin remains in the stomach, its sole site of production.

When the esophageal epithelium is exposed to acids, bile salts, and pepsin, it stimulates the secretion of proinflammatory cytokines such as interleukin-1, 6, 8, 10, and tumor necrosis factor-*α*. This contributes to the proliferation of T cells and neutrophils, resulting in chronic inflammation, oxidative stress, and proliferative activity (15). Tissue damage can occur from the inflammatory responses triggered by reactivated pepsin during a new reflux episode when pH falls below 6.0 (16). Additionally, these inflammatory processes generate oxidative stress and accumulate free oxygen radicals, which may ultimately damage mitochondria and lead to cell death (8). Moreover, toxic refluxate, such as pepsin, can also enter cells through the basolateral membrane, causing intracellular acidification and further cell damage (17).

Patients with GERD often exhibit impaired integrity of the esophageal mucosa, making them more sensitive to the harmful effects of pepsin and gastric acid. In recent years, the focus of research on this mechanism has gradually shifted from the apparent symptoms to the intrinsic molecular mechanism. The research progress of this mechanism focuses on the cellular pathway of chronic inflammatory response and the cytokines involved. According to several studies, the participation of matrix metalloproteinases (MMPs) in this pathogenesis are highlighted, especially MMP-1, 2, 3, 7, 9, 14 (18–20). For instance, exposure to gastric acid increases ROS and phosphorylates ERK1/2, ultimately leading to the phosphorylation of c-Jun. This signaling pathway is associated with increased expression of MMP-7 and the degradation of E-cadherin (20). By degrading E-cadherin, acid-activated pepsin disrupts epithelial integrity (18, 21). At the same time, reflux pepsin can also induce the expression and excessive proliferation of MMP as sheddase of E-cadherin (22). Additionally, the acidic gastric juice could also contribute to the elevated expression of MMP-9, further causing the degradation of occludin, a significant protein that constructs endothelial tight junctions (23). Inspired by this and supporting evidence from PubMed and Embase databases, we can conclude that the degradation of tight junctions (e.g., Claudin-1, 2 and 4, ZO-1, filaggrin, and occludin) induced by pepsin and gastric acid is another significant GERD pathology (24).

Studies have shown that the acute stress caused by acid and pepsin exposure can widen the epithelial tight junctions of the esophageal mucosa, increasing its permeability (25). More critically, through the study of animals esophageal epithelial tissue, Ergun et al. found that epithelial cells are more sensitive to chronic inflammation caused by harmful substances than acute inflammation. This provides a more accurate focus for clinical pathologic mechanism investigation (26–28). This allows acid and pepsin to penetrate the epithelial cells, leading to tissue necrosis (25). Dilated intercellular spaces (DIS), a common ultrastructural lesion in the basal layer, may result from inhibited sodium transport. This condition is attributed to the erosive effects of acid, bile, or pepsin. DIS promotes luminal ion flow, activating epithelial and neural receptors, which can ultimately lead to GERD (29). Consequently, this increases the risk of Barrett’s esophagitis and may lead to esophageal adenocarcinoma (21).

**Figure 1:**
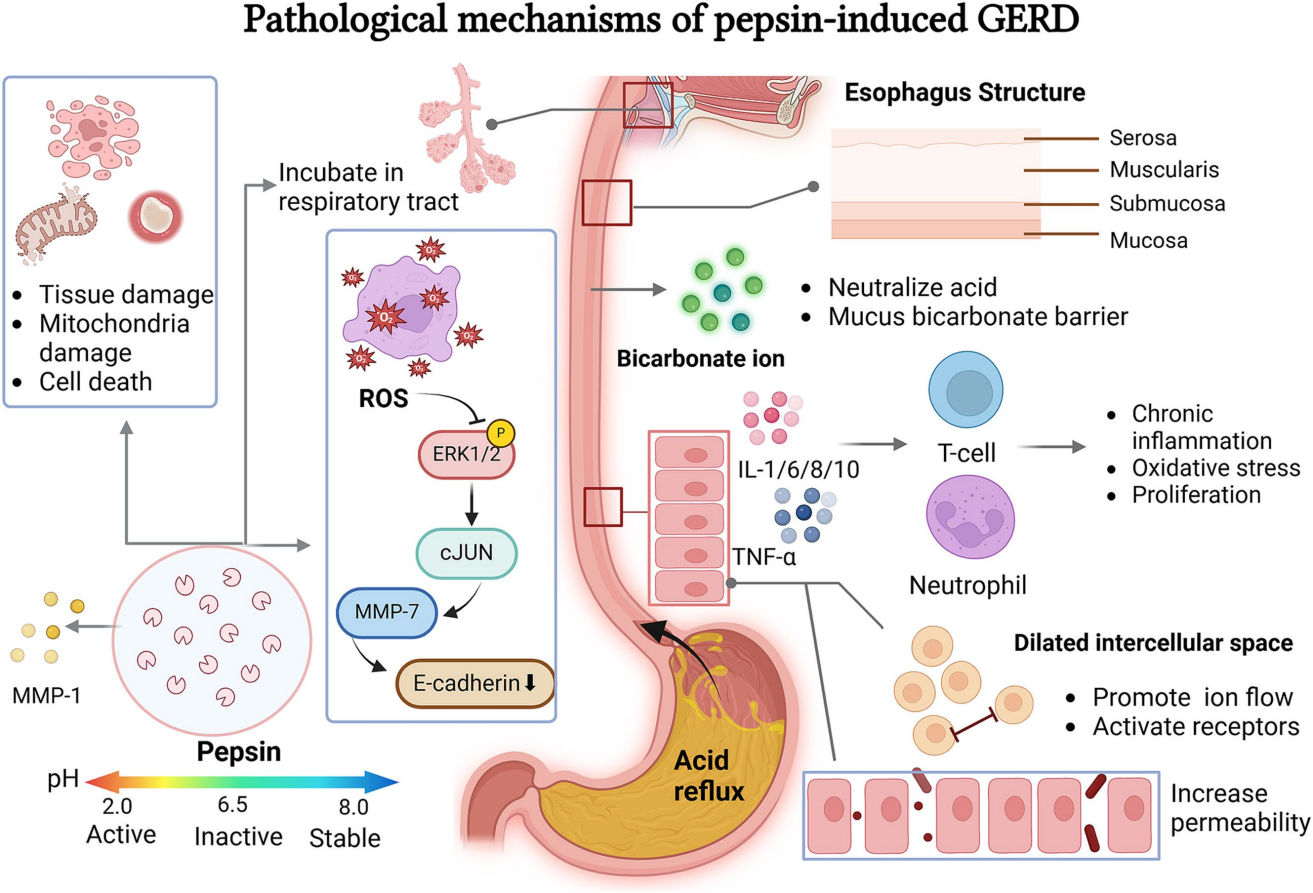
Pepsin related pathological mechanisms leading to GERD. Pepsin invades the esophageal wall and laryngopharynx through a series of pathological mechanisms. It activated ROS in the appropriate pH environment which can cause cell and tissue damage, ROS can also regulates the ERK-cJUN-MMP pathway to affect intercellular space by cleavage E-cadherin. The mucus bicarbonate barrier protects against the invasion of gastric acid and pepsin. After stimulation, esophageal epithelial cells activate a chronic inflammatory response, changing the activity and state of the cells and releasing regulation factors.

After passing through the esophagus, pepsin generated by reflux can bind to the respiratory tract mucosa and remain temporarily inactive after being neutralized by saliva and bicarbonate. As the most significant component of refluxate, pepsin can be found in the upper digestive tract, where it acts as a pathological agent contributing to the primary symptoms of GERD (13). Based on the aforementioned pathological mechanisms of pepsin, increased paracellular permeability in the esophageal epithelium may plausibly explain the heartburn symptoms in patients (29). At the same time, the stimulated esophageal epithelium produces an inflammatory response dominated by T lymphocytes, which also increases the sensitivity of the nerve endings located in the mucosa (23). The penetration of pepsin through the lamina propria to the visceral nerve endings can also contribute to these heartburn symptoms (17) (Figure 1).

## 3 Diagnosis of GERD

### 3.1 Background of pepsin detection

GERD is associated with various factors that lead to the reflux of acidic components into the esophagus, causing symptoms and complications (30). Clinical evaluations of symptoms form the foundation for diagnosing GERD, but diagnostic tests can either support or contradict the initial clinical assessment depending on the specific criteria used in each test. Therefore, understanding the strengths and limitations of each diagnostic tool and gathering multiple pieces of evidence is essential (31). The ACG clinical guidelines and various consensus documents provide conclusive diagnostic criteria for GERD, as well as recommendations on ruling out the condition.

**Figure 2:**
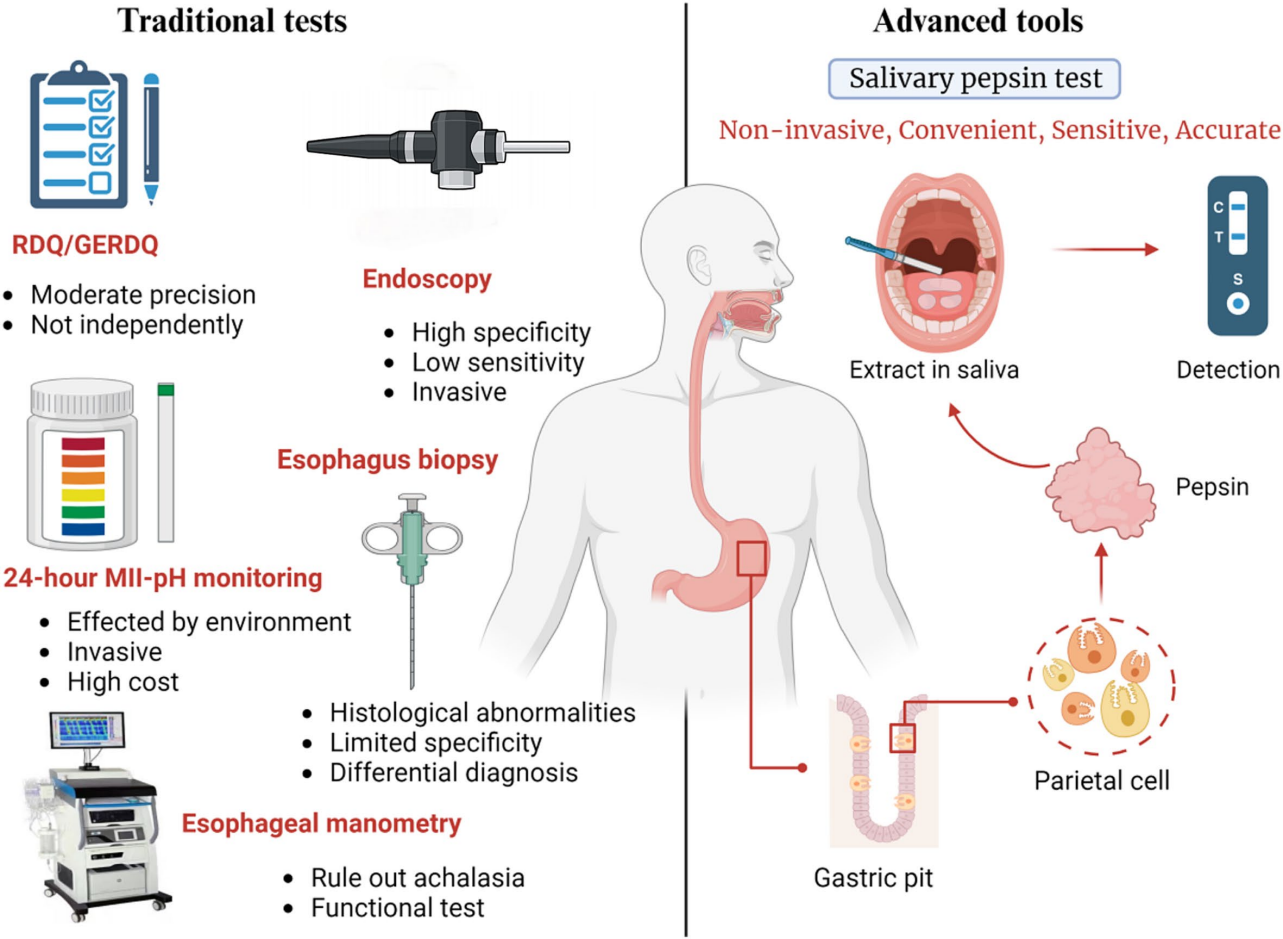
Introduction of diagnostic method with traditional test and pepsin detection in saliva. The traditional diagnosis consists of RDQ and GERDQ, endoscopy, 24H-pH monitoring, esophageal manometry and pathological biopsy. They all have some disadvantages including invasiveness, instability of results, and high cost. Detection of pepsin in saliva is expected to become widely used measurement by avoiding these problems while has high specificity and sensitivity.

At the moment, GERD can be diagnosed with specially designed questionnaires, proton pump inhibitors (PPIs) for anti-secretory therapy, endoscopy, and reflux monitoring tools (32–34). However, both the Reflux Disease Questionnaire (RDQ) and the GERD Questionnaire (GERDQ) have demonstrated only moderate accuracy (approximately 65–70%) and were specifically designed to identify symptomatic GERD. As a result, questionnaires alone are insufficient for a precise diagnosis (35). Additionally, while endoscopy has high specificity in diagnosing GERD, its sensitivity is quite low. Data shows that over 70% of GERD patients have normal esophageal mucosa on endoscopy, and one study even reported a normal mucosal rate of up to 90% in patients treated with PPI therapy (33, 36). As for pathological testing, reflux esophageal biopsies have limited specificity, and there is often disagreement among pathologists (32–34). Currently, esophageal function tests, such as 24-h MII-pH monitoring, are considered the most reliable diagnostic methods (37). However, these measurements cannot distinguish rumination or supragastric belching from the reflux episodes, and have a low sensitivity for counting reflux episodes accurately (32). Additionally, dietary restrictions and reduced physical activity during reflux monitoring may lead to false-negative results. Moreover, this test is expensive and invasive (38) (Figure 2).

### 3.2 Clinical salivary pepsin test

Traditional symptom-based diagnostic tests for GERD not only have limited sensitivity and specificity but are also costly and invasive. Therefore, there is a pressing need for the development of a clinically applicable, non-invasive, convenient, sensitive, and accurate diagnostic tool. Recent evidence has identified salivary pepsin as a promising biomarker for GERD, as this enzyme is recognized as a major contributor to the condition (5, 39). The diagnostic value of Peptest stems from the fact that pepsin is synthesized exclusively in the stomach, making its presence in the esophagus or other proximal structures a clear indicator of reflux (39). However, since salivary pepsin concentrations rapidly decrease after a reflux episode, samples should be collected as soon as possible to avoid enzyme degradation. Another method to improve the accuracy of results is to collect multiple salivary samples throughout the day (40).

A prospective study conducted in China evaluated the diagnostic utility of salivary pepsin and found that GERD patients had significantly higher pepsin concentrations in their saliva compared to a control group. Additionally, post-meal samples collected during symptomatic episodes in GERD patients showed higher pepsin levels and a greater rate of positive results compared to general postprandial samples. Using a cut-off value of 76 ng/mL, Peptest demonstrated a sensitivity of 73.0% and a specificity of 88.3%. The study also suggested that pepsin, as a significant marker of reflux, is strongly correlated with lower esophageal sphincter (LES) motility in GERD patients (39).

In another study validating the diagnostic utility of the salivary pepsin test in China, the overall sensitivity and specificity for both genders were found to be 85 and 60%, respectively. The patients involved in the study had been pre-diagnosed using questionnaires and invasive endoscopic examinations. Beyond its high accuracy, this novel non-invasive test offers the advantage of providing rapid diagnostic results for GERD (41). In urgent situations, Peptest can also be used to differentiate GERD-related chest pain from acute coronary syndrome (ACS), which shares similar symptoms. In such cases, Peptest demonstrated a positive predictive rate of 90% and a negative predictive rate of 62%, helping to avoid unnecessary cardiological evaluations (42).

Additionally, it has been shown that the salivary pepsin test can help distinguish between extra-esophageal symptoms and respiratory disorders, as well as between typical GERD symptoms and reflux hypersensitivity. This reduces the risk of misdiagnosis and delayed treatment, thereby improving patient prognosis (38, 43).

More recently, a prospective study examining the relationship between salivary pepsin levels and endoscopically confirmed EE demonstrated that Peptest has excellent sensitivity and a high negative predictive value in diagnosing EE, a pathological condition associated with GERD (44).

### 3.3 Latest fluorescence detection of salivary in lab

While the studies mentioned above demonstrate the potential of salivary pepsin tests with significant sensitivity and specificity for GERD diagnosis, their therapeutic application remains a topic of debate. The variability in salivary pepsin concentrations among individuals makes the test less suitable for consistent clinical use (45). Therefore, it is crucial to promptly collect salivary samples with a predetermined threshold of salivary pepsin concentration to achieve a more accurate diagnosis of GERD (46).

Improving the accuracy of pepsin detection thresholds in saliva is necessary due to the complexity of the salivary environment, which contains a variety of interfering proteins and compounds. Fluorescence detection has gained widespread use because of its exceptional efficiency, convenience, speed, specificity, and strong resistance to interference. Several studies have shown that using fluorescence detection to measure salivary pepsin concentrations can be a fast and precise method for screening GERD (47–49).

#### 3.3.1 Strong electrostatic interactions

Strong electrostatic interaction-based fluorescence detection is a method that relies on the interaction between pepsin and SYBR Green (SG) fluorophores (50). Pepsin has a low isoelectric point and carries a negative charge, making it prone to binding with positively charged SG fluorophores at neutral pH. Upon adsorption, the rotation of SG molecules is restricted, leading to a significant increase in fluorescence intensity. Based on this principle, researchers have developed a fluorescence capture device, integrated with Python programming, that enables the precise detection of pepsin in less than three minutes. This assay is highly specific, simple, and cost-effective, with a detection limit of 0.2 μg/mL (47).

#### 3.3.2 Colorimetric dipstick assay

The pepsin-sensitive peptide (PSP) consists of two amino acids that are specifically cleaved by pepsin, along with eight amino acids that remain uncleaved (48). Fluorescein isothiocyanate (FITC) and biotin are used as reporter genes for dipstick colorimetric detection, with modifications made to the N-and C-terminals. The efficiency of pepsin degradation of PSP is assessed by analyzing the fluorescence of FITC. When PSP reacts with pepsin in the test tube, both remain colorless on the test line. Consequently, there is an inverse relationship between the pepsin concentration and the ratio of the color intensity of the test line to the control line (IT-line/IC-line). After conducting multiple experiments, researchers determined that cutting reactions on test paper at 42°C for 30 min provides optimal pepsin detection. Additionally, a propylene filter is used to pre-treat saliva in point-of-care testing (POCT). The dipstick method has demonstrated superior sensitivity compared to ELISA, which serves as a reference standard (48).

#### 3.3.3 Bovine serum albumin and squaraine dye assembly fluorescent probe

Squaraine dye (SQ) is a fluorescent dye that exhibits strong absorption and fluorescence emission in the near-infrared range, and it has a distinct aggregation-caused quenching (ACQ) effect. Bovine serum albumin (BSA) interacts with SQ through hydrophobic and hydrogen bonding, forming BSA-SQ assemblies that generate hypofluorescence in a Gly-HCl solution (51). Pepsin hydrolyzes BSA, increasing the exposure of SQ in the system, which results in a reduction of the fluorescence emitted by the probe. The detection of the “switch on/off” change in fluorescence signal enables fluorescence-based analysis of pepsin. The optimal concentration of BSA for this assay is 15 μM, with the reaction conducted at a pH of 2.6 for 25 min, producing the most sensitive response. This probe exhibits exceptional sensitivity, selectivity, and a wide detection range, making it ideal for quantitative analysis of pepsin (52).

#### 3.3.4 Supramolecular tandem assay

The supramolecular tandem assay (STA) can be used as an indicator displacement assay (IDA) with a host-guest reporter pair for signaling, allowing it to monitor enzymatic activity (53). Recent reports describe the use of a calixarene-based STA strategy for determining pepsin concentration. In this method, lucigenin (LCG) and p-sulfonatocalix[4]arene (SC4A) are chosen as the supramolecular reporter pair, while insulin is used as the enzymatic substrate, as it is susceptible to hydrolysis by pepsin at pH 2.0. The SC4A-insulin complex interacts with LCG, triggering a fluorescence reaction. When insulin is degraded by pepsin, the fluorescence signal is inhibited (54).

Compared to conventional diagnostic procedures for GERD, the STA strategy offers several advantages, including convenience, non-invasiveness, comfort, and low cost. Additionally, pretreatment steps are unnecessary, and its point-of-care testing (POCT) potential increases commercialization prospects. However, the assay’s sensitivity limits its ability to detect low concentrations of pepsin in saliva.

#### 3.3.5 Magnetic molecularly imprinted nanoparticle assay (MINA)

Fluorescent pepsin-specific molecularly imprinted polymer nanoparticles (nanoMIPs) are used in this assay. Magnetic pepsin nanoparticles (MPNs) are immobilized on magnetic microtiter plate inserts via fluorescent pepsin-specific nanoMIPs (55). After the imprinting process, the nanoparticles are modified with the commercially available fluorophore AlexaFluor® 647 NHS ester to improve the sensitivity of the MINA. The competition between free and immobilized pepsin leads to fewer nanoMIPs binding to the magnetic insert, resulting in an increase in fluorescence intensity. This approach reduces both the time and cost of the assay while avoiding the use of antibodies, thereby eliminating the need for animal-derived reagents (56).

#### 3.3.6 Lysozyme-stabilized au nanoclusters

Fluorescent metal nanoclusters offer several advantages, such as their small size, good biocompatibility, and excellent photostability, making them ideal for biological applications as fluorescent markers (57). Lysozyme reacts with AuCl4-at 37°C and pH 3.0 for 3 h, forming the AuNC@Lyz complex, which induces a fluorescent response. The specific enzymatic interaction between the luminescent nanoclusters (AuNCs@Lyz) and pepsin results in the degradation of lysozyme and a decrease in fluorescence intensity. This method enables the detection of pepsin with both high sensitivity and selectivity. It exhibits a linear pepsin detection range from 1 mg/mL to 100 mg/mL and has a detection limit of 0.256 mg/mL (58).

#### 3.3.7 Electrochemical immunosensor

In recent decades, nanomaterials have emerged as promising substrates for developing innovative electrochemical biosensors due to their small size and efficient catalytic properties (59). Using a soft template synthesis method with *β*-naphthalenesulfonic acid (NSA), researchers fabricated polypyrrole nanocorals (PPNCs) on a screen-printed carbon electrode (SPCE). Gold nanoparticles (GNPs) were then electrochemically deposited onto the PPNCs/SPCE composite, followed by immobilization of pepsin on the GNPs. At each stage of the immunosensor process, the interaction between the antibody and antigen was monitored using cyclic voltammetry (CV). Results showed that the electrochemical immunosensor displayed high sensitivity in detecting pepsin (60).

#### 3.3.8 Carbon dots-protein interactions biosensor

According to a new study, scientists have discovered a highly specific and sensitive pepsin biosensor for detecting pepsin in saliva. As a new type of spherical carbon material with a size less than 10 nm, carbon point (CD) is simple to prepare, low cost, easy to modify, good hydrophilicity, and good fluorescence stability, and has been widely used in production as a new material in recent years (61–63). This method is based on the coupling principle of green-emitting ionic liquid-based carbon dots and whey proteins, which interact to form an aggregation structure of G-IL-CDs as a high-performance fluorescent probe (49). When the concentration of pepsin increased, the structure was destroyed and the fluorescence concentration was changed. The concentration of pepsin can be quickly, cheaply and non-invasively assessed by the detected fluorescence intensity (49).

## 4 Management of GERD

### 4.1 Proton pump inhibitor (PPI)

Proton pump inhibitors (PPI) are the first-line treatment for GERD and effectively reduce gastric acid secretion and the acidity of the stomach contents (46). Additionally, they inhibit the conversion of pepsinogen into pepsin, protecting the esophagus from damage similar to mucosal lesions caused by pepsin (64). However, drug use is accompanied by tolerability issues, including discontinuation for any reason, (ineffectiveness, adverse effects, and noncompliance). Numerous studies have shown that PPI use can lead to a range of side effects and long-term complications (65–67). CYP2C19 polymorphisms can affect the metabolism rate of PPI, and patients with CYP2C19 mutations have poor response to PPI (68). In addition, PPI only reduces reflux acidity but not frequency and increases the concentration of pepsin and bile (46). Long-term use of PPI with high compliance may increase the risk of esophageal adenocarcinoma (69). Meanwhile, the alternation of pepsin concentration caused by PPI can produce pathological consequences. Experiments have found that patients exposed to acid suppression are more likely to present inflammatory cytokine secretion, barrier disruption and neutrophil migration in gastric juice compared with patients not taking PPI (70). To improve efficacy, new drugs such as potassium-competitive acid blockers (P-CABs) have been developed as alternatives to PPI.

### 4.2 Mucosal protective agents

Mucosal protectants have been regarded as another important means for the treatment of GERD and LPR in recent years, and their main effect is to control the occurrence of inflammation in the epithelial tissue and maintain the integrity of the mucosa.

As the most commonly used and studied mucosal protective agent in clinic, Alginates are polysaccharide polymers that form a viscous, low-density gel upon contact with gastric acid (71). To displace the postprandial acid pocket and inactivate pepsin at the gastroesophageal junction, alginates create “rafts” on the surface of the stomach contents, preventing pepsin from reaching the esophageal wall and thus reducing GERD incidence. At the same time, alginates adhere to the esophageal mucosa, shielding it from barrier disruption and cell detachment caused by prolonged exposure to high concentrations of pepsin and acid (72). Additionally, alginates have antioxidant and anti-inflammatory properties that help neutralize ROS and cytokines (18). They also protect epithelial integrity by inhibiting the expression of MMP produced by pepsin. GERD patients treated with alginates had fewer acid pockets compared to those treated with traditional antacids. Approximately 71% of these patients showed acid pockets positioned below the diaphragm, which is negatively correlated with acid reflux (72). At the same time, meta-analysis results of randomized controlled trials have shown that alginate preparation in GERD patients has better efficacy than PPIs or controls, and some known adverse reactions of PPI are avoided. It is necessary to continue to explore the feasibility of its replacement for PPI in future clinical trials (73).

In addition, we also summarized other macromolecular polymers used in GERD therapy in recent years. For example, clinical studies have found that bio-polymer of cashew gum and polysaccharide of *Gracilaria caudata* have the function of mucosal protective activity in human esophageal biopsy (74, 75). As a new material with anti-microbial, anti-inflammatory, pro-healing pharmacological properties, Angico Gum (Anadenanthera colubrina) biopolymer was found to be both anti-inflammatory and protect the integrity of esophageal mucosa in mouse models (76, 77).

### 4.3 Protease inhibitors

Nagaham et al. previously reported the effectiveness of pepstatin A in reducing inflammation and fibrosis, as well as its role as a pepsin inhibitor in preventing esophageal ulcers in experimental models of esophagitis (6). Building on this, scientists have observed that the administration of aspartic protease inhibitors, particularly darunavir and fosamprenavir, in animal models of laryngeal reflux disease (LPR) has shown remarkable protective effects on the mucosal barrier and has inhibited laryngeal inflammation (78). Amprenavir, a derivative of fosamprenavir, functions as an HIV protease inhibitor and exhibits a protective effect against pepsin-induced esophageal epithelial barrier disruption and cancer-related changes by inhibiting pepsin at lower doses (79). This process helps preserve the integrity of the laryngeal epithelium and prevents pepsin-induced damage to cell adhesion molecules at pH 4 (79, 80). Additionally, amprenavir partially rescued pepsin-mediated E-cadherin cleavage and suppressed pepsin-induced upregulation of MMPs (79). Phase I clinical trials have tested fosamprenavir via dry powder inhalation (DPI) to reduce the risk of severe side effects associated with high doses and to improve treatment efficacy. This method allows for localized administration of modest doses in powder form. However, the optimal diameter of the inhaled powder particles has yet to be determined (81).

The clinical potential of pepsin inhibitors is limited by their poor solubility, particularly in water. Selecting appropriate solvents to increase the solubility of pepsin inhibitors without reducing their effectiveness is a significant challenge for researchers. Additionally, structural data suggest that inhibitor binding to pepsin is primarily stabilized by van der Waals interactions, making the design of effective inhibitors more difficult. As a result, further exploration of other aspartic protease inhibitors is needed to improve therapeutic outcomes. Studies that provide comprehensive pre-and post-treatment data, along with collaborative efforts, are essential to predict clinical success.

### 4.4 Prospective future

Though the above-mentioned studies on salivary pepsin test with high sensitivity and specificity have a promising future in diagnosing GERD, the clinical utility of peptests is still under debate need to be progressed. To eliminate biases, the collection of salivary samples at post-symptomatic time with a determined threshold of salivary pepsin concentration should be urgently utilized for a better diagnosis of the GERD (45). Additionally, larger-scaled studies should be conducted in order to alleviate the occasionality of studies within small groups. More importantly, the collaboration of data from both pre-and post-treatment are highly needed to predict reliable clinical outcomes and verify the peptests’ diagnostic efficiency (39). At the same time, the market is in urgent need of highly sensitive and specific measurement tools to improve the diagnostic efficiency of GERD. Although a variety of new fluorescent probes made of biomaterials have been discovered, there is still a long way to go before clinical application. In addition, oral soft tissue disorders and the detection of some specific oral microorganisms in dental erosions (DE) and periodontal diseases (PD) is also found to be useful for differential diagnosis of GERD (82, 83). Moreover, there are studies that reveals higher BMI is relevant to higher incidence of GERD, which can be a supplementary information for accurate GERD diagnosis (84). Unfortunately, there are few studies on the association research, so we cannot draw objectively complete verified conclusions.

Except beyond the newly developed treatments, preserving the barrier function and maintaining the integrity of the esophageal epithelium are critical factors in defending against GERD. Studies have shown that mice possess stem cells in the basal layer of the esophagus that can differentiate into superbasal cells, aiding in the repair of the esophageal epithelium after injury (85). In future therapeutic approaches, it may be possible to stimulate the migration of squamous epithelium toward columnar epithelium to enhance resistance to gastric acid. Additionally, stem cell transplantation to reconstruct damaged areas of the esophagus is a promising treatment option. Pepsin, when acidified, can degrade key cell surface proteins, including those involved in cell–cell junctions. Therefore, another potential area of research is targeting adhesion molecules such as E-cadherin to promote their expression in the esophageal epithelium. This strategy could help counteract the acidifying effects of pepsin and preserve the structural and functional integrity of epithelial cells. The preventive effect of a 30% ethanol extract from the rhizome of *Curcuma longa* (CLR) on acute reflux esophagitis (ARE) caused by GERD has been attributed to its ability to enhance antioxidant factors, thereby reducing inflammation (86). Gel-type mucosal protective agents based on macromolecular materials will be a promising therapeutic method.

According to the latest Lyon Consensus 2.0, endoscopy, wireless pH monitoring, catheter-based 24 h pH or pH impedance monitoring, and high-resolution esophageal manometry performed during the absence of anti-acid secretion therapy are considered to be highly effective methods for the diagnosis of GERD. The consensus also suggests that long-term wireless pH monitoring is the preferred diagnostic tool, and endoscopy performed 2 to 4 weeks after cessation of PPI testing can maximize diagnostic accuracy (32). Though salivary pepsin test cannot completely replace the above-mentioned diagnostic tests, it can be an effective supplementary tool for the diagnosis of GERD. According to the collected and summarized relevant information, despite its limitations, the salivary pepsin test is less invasive and more cost-effective compared to traditional methods. Future studies should focus on addressing its shortcomings and validating its clinical utility. Moreover, with the advancements in precision medicine and improving healthcare standards, there is an increasing demand for rapid, non-invasive, and side-effect-free management options for GERD. The use of targeted pepsin in both the diagnosis and treatment of GERD holds substantial clinical value and potential.
